# Assessing the feasibility of indoor air quality monitoring in low-income housing among Black adults with uncontrolled asthma in New York City

**DOI:** 10.1186/s12889-026-28295-z

**Published:** 2026-07-08

**Authors:** Maureen George, Rhea K. Khurana, Jean-Marie Bruzzese, Marija Zeremski, Emily DiMango, Aisha Naseem, Andrea Cassells, Eunice Mak, Natalie Benda, Jonathan N. Tobin, Steven Chillrud

**Affiliations:** 1 Columbia University School of Nursing, 630 West 168th Street Mail Code 6, New York, NY 10032 United States; 2 Clinical Directors Network, Inc. (CDN), New York, NY United States; 3 Columbia University Irving Medical Center, New York, NY United States; 4 Center for Clinical and Translational Science, The Rockefeller University, New York, NY United States; 5 Lamont-Doherty Earth Observatory, Columbia University, New York, NY United States

**Keywords:** Home environmental audit, Particulate matter, Respiratory health disparities, Urban health

## Abstract

**Background:**

Indoor air quality (IAQ) plays a critical role in respiratory health, particularly among individuals with asthma. This study evaluated the feasibility of collecting and characterizing IAQ in the homes of Black adults with uncontrolled asthma in New York City.

**Methods:**

Home environmental audits were conducted at the time that ultrasonic personal air sampling (UPAS v2+) devices were deployed, which continuously collected both time-resolved and integrated air samples over an initial target runtime of two-weeks based on the amp-hours of the external battery. Post-trial interviews were conducted.

**Results:**

Thirty devices were successfully deployed and returned; all but five provided sufficient data for analysis for getting a multi-day exposure estimates defined as more than 6 consecutive days of data) for analysis. Average runtime was 13.4 days and mean gravimetric PM_2.5_ was 72 µg/m^3^ which is double the Environmental Protection Agency’s cutoff of 35.5 µg/m^3^, a 24-hour average used to assess exposure and risk. Exposure to household environmental triggers was common. Study procedures were acceptable to participants, demonstrating the feasibility of collecting indoor air samples in apartments of adults with uncontrolled asthma.

**Conclusions:**

Elevated indoor PM_2.5_ levels and frequent exposure to environmental triggers were common and may contribute to poor asthma control.

**Trial registration:**

This feasibility study was conducted in NYC, New York, as an administrative supplement to a randomized controlled trial (RCT) (#NCT05685381; registered 1/5/23).

**Supplementary Information:**

The online version contains supplementary material available at https://doi.org/10.1186/s12889-026-28295-z.

## Background

Increasing evidence identifies fine particulate matter (PM_2.5_), nitrogen-based air pollutants, and ozone (O₃), as hazardous to human health, primarily due to their role in driving airway inflammation and hyperresponsiveness. Fine particulate matter (PM_2.5_), which consists of hundreds of chemical constituents, can penetrate deep into the lungs and represents one of the greatest risks to respiratory health [1]. 

Indoor air quality (IAQ) is particularly important to health as 90% of time is spent indoors [2] and remediation can be effective [3]. Methane, a potent greenhouse gas (GHG), is the primary component of natural gas [4] and gas appliances are the main residential source of PM_2.5_ [5]. While appliances can be vented outdoors, gas stoves are largely unvented. More than 40% of all homes in the United States use either natural gas or propane as their stove’s fuel source [6], with use in New York City (NYC) being much higher (> 62%) [7, 8]. Those most vulnerable to indoor air pollution are those who live in homes with gas appliances that are inadequately vented [9]. Although NYC enacted a natural gas ban for new housing in December 2021 [10], this will have little effect on the older housing stock that dominates NYC residential living spaces. Older apartment buildings commonly feature kitchens that open directly into living areas and lack properly functioning, externally vented range hoods, which may contribute to poor indoor air quality. Combustion of natural gas, particularly in the presence of other combustible sources such as environmental tobacco or marijuana smoke, incense or candle burning, and the use of aromatherapy diffusers generates a range of indoor air pollutants. Public housing apartments frequently experience additional indoor air quality challenges—including inadequate ventilation, moisture and mold, pest infestations, environmental tobacco smoke and pollutant transfer between units—all of which may exacerbate asthma symptoms [11, 12]. Indoor pollutants may also originate from outdoor sources that infiltrate the home through open windows or building leakage [12]. 

Individuals with respiratory conditions such as asthma are particularly susceptible to adverse health effects from poor indoor air quality (IAQ), which may be exacerbated by gas stoves and other indoor environmental triggers. Poor IAQ is a well-documented cause of suboptimal pediatric respiratory health [12, 13]. However, less is known about the impact of poor IAQ on adults with uncontrolled asthma.

Asthma is a common chronic condition affecting more than 20 million American adults. Those living below the poverty level have higher asthma prevalence relative to those living above the poverty level [14]. Asthma is also more common in adult women (9.7%) compared to adult men (6.2%) [14] which may explain, in part, the higher rates of primary care visits for women (450.5 per 1,000) compared to men (272.4 per 1,000) [15]. Relative to non-Hispanic White and Hispanic adults, Black adults have the highest asthma prevalence (8.5% v. 11.5% v. 12.8%, respectively) [14]. While nearly 40% of all adults with asthma have uncontrolled disease [16], more than 90% of those with severe asthma have uncontrolled asthma [17]. This leads to high ED visit rates of adults with asthma (27.8 per 10,000 individuals) [16]. Black adults experience more severe disease and higher rates of uncontrolled asthma compared to other racial and ethnic groups [18–20]. With this greater disease burden, it is unsurprising that Black adults die from asthma at a much higher rate than White and Hispanic adults (22.7 v. 8.1 v. 7.1/million, respectively) [16]. Adults also die from asthma at nearly six times the rate of children with asthma [21]. 

Despite higher asthma prevalence and mortality among adults, prior IAQ studies have largely focused on children’s indoor allergen and environmental tobacco smoke exposure in homes and schools [22–33]. With comparatively less attention to PM_2.5_ exposure from cooking stoves in apartments with open floor plans, our ongoing trial of Black adults residing in this community (#NCT05685381) provided a unique opportunity to collect data in a vulnerable but understudied adult population while simultaneously addressing feasibility questions about potential barriers to home access i.e., whether adults would permit entry and whether deploying and retrieving monitoring equipment in low-income housing would be feasible. Therefore, the primary aim of this feasibility study was to sample and characterize the team’s ability to collect IAQ data in low-income NYC residences of Black adults with uncontrolled asthma.

## Methods

### Study location

This feasibility study was conducted in NYC, New York, as an administrative supplement to a randomized controlled trial (RCT) (#NCT05685381).

### Recruitment

Participants were recruited if they had participated in a pre-trial interview or were screened and determined to be eligible for participation in the parent RCT. Eligibility criteria included (1) aged 18 or older (2), self-identified Black race (African American (AA), Black or AA/ Black and Hispanic or other race) (3), uncontrolled asthma as measured by an Asthma Control Questionnaire [ACQ] score ≥ 1.5 [34], and (4) receiving asthma care at a Federally Qualified Health Center (FQHC) in NYC that are members of Clinical Directors Network (CDN) primary care practice-based research network (PBRN).

### Methods

The study components included a two-week period of home IAQ monitoring, an environmental audit of indoor triggers, and a post-monitoring interview.

#### UPAS v2 + deployment

An Ultrasonic Personal Air Sampler v2+, mounted on a camera tripod, (UPAS v2+; Access Sensor Technologies, Fort Collins, CO) was deployed by study personnel in the primary living area (e.g., living room, bedroom, or kitchen) of subjects’ apartments, at a height of 3 feet and a minimum of 1 foot from any wall, provided a working electrical receptacle was available in the designated location. The UPAS v2 + continuously estimates PM_2.5_ concentrations using a light-scattering optical sensor and includes an integrated filter that allows calibration of the continuous measurements to the gravimetrically derived integrated PM_2.5_ levels [1, 35], a well-validated method for measurement [1]. Earlier prototypes of the UPAS monitor have been validated for PM_2.5_ [36–38]. The UPAS v2 + is a lightweight, cell-phone sized unit that is easier to deploy and maintain compared to earlier models. An accelerometer in the UPAS v2 + can determine activity level and location compliance (e.g., the device’s movement after deployment). Low-cost gas sensors within the UPAS v2 + provide nominal responses to other components of indoor air (i.e., carbon dioxide (CO_2_), nitrogen oxides (NOx), and volatile organic compounds). CO₂, NOx, and VOCs are not reported here due to distinct measurement and interpretive challenges (e.g., CO₂ lacks a health-based ambient standard and functions more reliably as a ventilation proxy than a pollutant exposure metric); NOx measurement is subject to baseline sensor drift that complicates data analysis and validity; the VOC sensor is prone to both sensor baseline drift and cross sensitivity to other parameters.

A combination of plug-in and battery-powered units were used in this study. For both unit types, a 3-D printed holder was constructed to secure each UPAS v2 + upright, protecting it from accidentally spilled liquids and concealing the on/off button. The plug-in units were susceptible to being unplugged by participant family members or losing power. For the battery-operated unit, a plastic box enclosed a 67 AmpHr external battery and charging cables to extend the runtime of the monitor whose internal battery of 6.7 AmpHr allows 48-hour run time at 20% duty cycle on the filter; and as such we thought the external battery would be more than sufficient for a 14-day deployment.

After the first several deployments of units with the external battery only ran for 8 days, thus failing to meet the 2-week target runtime, we modified the battery system to allow it to also be plugged into line power so that it could charge the external battery while the battery was also powering the UPAS v2 + air monitor. The modified unit functioned with its external battery as an uninterruptable backup power supply, capable of sustaining up to 8 days of operation in the event of power interruption from any cause (e.g., accidental disconnection from the wall outlet, utility power outage). For data analysis, a minimum deployment duration of 6 days was established as the acceptance criterion, sufficient to demonstrate feasibility, generate a multi-day average concentration, and yield a valid filter net weight.

#### Home environmental audit

A home environmental audit, adapted from the NYC Neighborhood Asthma and Allergy Study [39], was conducted at the time of UPAS v2 + deployment and included a visual inspection of the residence to document the presence and type of stoves and ovens (gas or electric), their functionality, and the presence of exhaust fans or range hoods. An investigator-developed survey included objective assessments of the home conducted by the research staff and self-reported answers to staff administered questions, such as self-reported frequency of gas stove use, presence of environmental tobacco smoke (ETS), use of aromatherapy diffusers, incense or scented candles, and the location and opening of windows facing major or minor roadways (see Supplemental Table 1).

#### Post-monitoring interview

A semi-structured interview (see Supplemental Table 2), guided by constructs from the Theoretical Framework of Acceptability—including burden, user experience, attitudes, and participation intentions—was conducted via a HIPAA-compliant Zoom dial-in conference call (audio-only) following UPAS v2 + retrieval to evaluate participant satisfaction with and acceptability of the IAQ monitoring protocol. All interviews were audio-recorded for analysis.

### Post-trial protocol amendment

Because several apartments had elevated PM_2.5_ levels, the study team implemented a post-trial protocol amendment to return results to participants, consisting of a grade-school reading level handout on free or low-cost strategies to improve IAQ, local and state remediation resources, as well as a prototype infographic illustrating their home air quality levels relative to established safe exposure thresholds.

### Quantitative analysis

Field blanks were taken when deploying devices in three participants’ homes to confirm that the samples were free from contamination. To confirm the precision of the samplers, duplicate samples were taken in three study team members’ homes. Filters from UPAS v2 + underwent gravimetric analysis using pre- and post-weighed filters to accurately determine the mass of particulate matter. The raw PM_2.5_ score was then multiplied by a gravimetric correction ratio, which is the gravimetric PM_2.5_ concentration based on the difference in weight of filter before and after sampling divided by the volume of air passing through the filter, divided by the average of the sensor-based PM_2.5_ [1]. The normal distribution assumption was checked, and non-parametric methods were applied to skewed distributions, therefore associations between PM_2.5_ levels and defined groups based on exposure to household environmental triggers (Yes/No groups) were tested using the Mann-Whitney *U* test.

### Qualitative analysis

All audio files were transcribed. Directed coding to identify constructs of the Theoretical Framework of Acceptability [40] was performed.

### Ethical review and compensation

The study was approved by WCG IRB (Tracking ID: 20211166). All participants provided informed consent in accordance with institutional review board–approved procedures. Participants received $40 at time of deployment and completion of the home environmental audit and $60 at the time of UPAS v2 + retrieval (total payment $100). Interviews were conducted using Zoom conference call feature initiated by either the study staff at the time of UPAS v2 + retrieval or by the research team within one week of retrieval.

## Results

### Sample

The sample consisted of 30 participants (90% female) with a mean age of 53 years (SD = 13, range 26–70). Mean Asthma Control Questionnaire (ACQ) score was 2.9 (SD = 0.93; scale = 0–6; uncontrolled asthma is ≥ 1.5). Participants also reported a mean of 6.4 inhalations of rescue medication in the week prior to enrollment and 37% reported one or more emergency departments in the prior year. See Table 1 for additional details.

**Table 1:** Characteristics of participants’ homes

	*N* (*n* = 30)	Percent (%)
Home Type		
Apartment	27	90
Brownstone	2	7
Single Family Home	1	3
Height from street (floor #)*		
0–2	10	33
3–5	9	30
6–8	8	27
Stove Type and Function		
Gas, working	22	73
Gas, not working	1	3
Electric, working	7	23
Electric, not working	0	0
Kitchen ventilation present (function not assessed)	15	50
Kitchen contiguous with living area	27	90
Windows facing minor arteries		
0–1	23	77
2–3	7	23
Windows facing major arteries		
0–1	15	50
2–3	7	23
4+	8	27
Years living in the home		
0–1 years	2	7
2–3 years	5	17
4+	23	76

*missing data

Twenty-seven (90%) participants lived in apartments and 10 (33%) lived in subsidized housing (New York City Housing Authority – NYCHA). NYCHA, who provides housing to economically disadvantaged households, also known as Sect. 8 housing, administers the largest Sect. 8 program in the United States with over 25,000 property owners and 177,565 apartments across 2,410 buildings [41].

### Feasibility trial

All participants completed all three components of the study: two weeks of IAQ home monitoring, a home environmental audit, and post-monitoring interview (100% retention).

#### UPAS v2 + performance

The goal was to collect a minimum of six days of usable data. At the start of the study, engineering units, powered by a wall socket, were the primary UPAS v2 + unit used for the study. The plug-in units were occasionally disconnected from power by the participants, leading to short runtimes and unusable data. Therefore, we modified the battery system as described in the Methods. Twenty-five units provided usable data and five did not (83% IAQ data collection completion). Only one participant required redeployment; the initial placement produced just 37 min of runtime, which was inadequate for sufficient PM_2.5_ filter collection and did not meet the ≥ 6-day threshold. The participant also requested a repeat deployment. Data from the second deployment only were included for this participant. The average run time for the UPAS V2 + units was 13.4 days.

#### IAQ sampling

Field blanks had a net mass change of 2.3 µg which represented 1.2% of the median mass of PM2.5 collected on the sample filters and less than 6% of smallest mass collected.

The average gravimetric PM_2.5_ levels from the 25 units providing usable data (median run time of 13.4 days) was 72.2 µg/m^3^ (SD = 46.1), well above the Environmental Protection Agency’s (EPA) 24-hour guideline value of 35 µg/m^3^ and 8 times higher than the annual average standard of 9 µg/m^3^ [42]. Using the EPA’s Air Quality Index as a guideline for interpreting 24-hour fine particulate matter exposure [42], a cutoff of 35.5 µg/m^3^ was selected to indicate poor indoor air quality for sensitive groups. Using this threshold, 15 participants (60%) had unhealthy household PM_2.5_ levels, with 3 of these homes having their deployment period average PM_2.5_ concentrations > 125 ug/m^3^ (placing them in the unhealthy or hazardous categories). Using the annual standard, 100% or all the participants’ homes had elevated levels. PM_2.5_ measurements categorized by EPA Air Quality Index levels are presented in Table 2.

**Table 2:** Household average PM2.5 categorized by EPA air quality index guidelines (*n* = 25)

PM_2.5_ range (µg/m^3^)	Levels of concern	*n* (%)
0.0–12.0	Good	2 (8)
12.1–35.4	Moderate	8 (32)
35.5–55.4	Unhealthy for sensitive groups	7 (28)
55.5-125.4	Unhealthy	5 (20)
125.5-225.5	Very unhealthy	2 (8)
225.5+	Hazardous	1 (4)

**Fig. 1:**
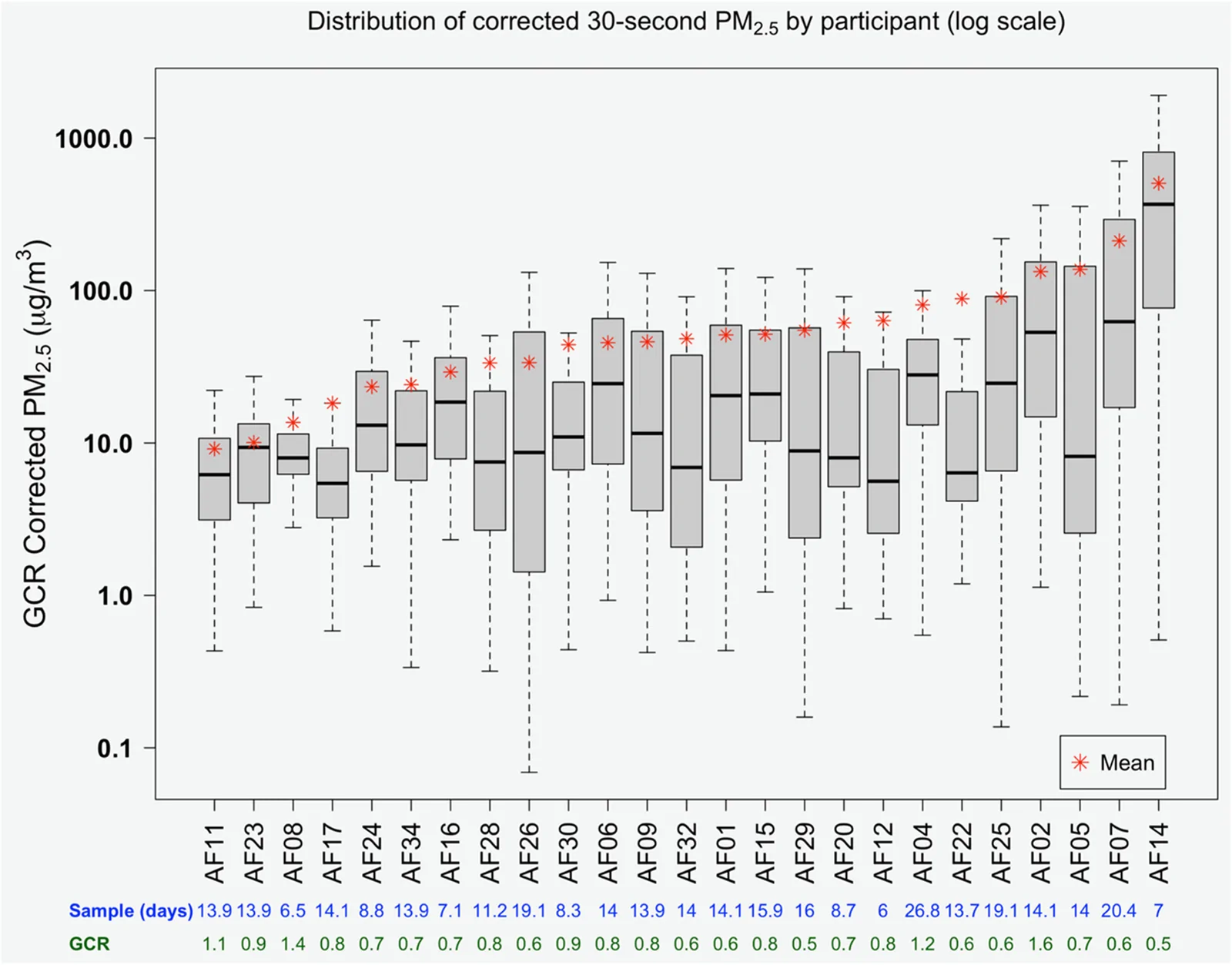
Distribution of gravimetrically corrected 30-second averaged sensor data for PM_2.5_ measured in each home, ranked by the mean corrected PM_2.5_concentration as indicated by red asterix. The mean is also equivalent to the gravimetric PM_2.5_ concentration measured over the entire deployment time period, which is shown at bottom of each box plot as well as the gravimetric correction ratio (GCR). Outlier points (not shown) are consistent with the log normal distribution that is apparent in most homes by the large offset between mean and median values

**Fig. 2:**
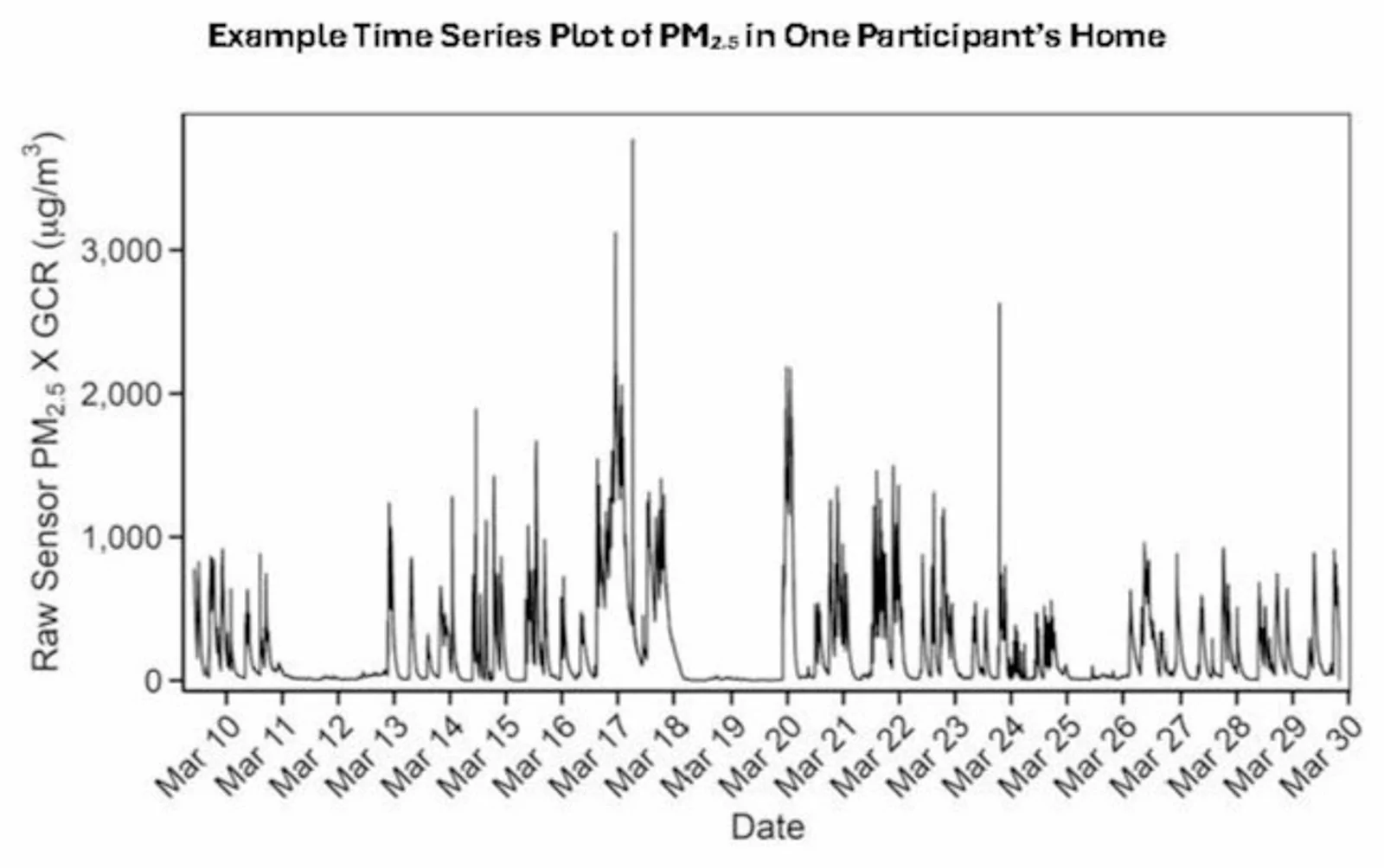
Time series of 30-second average concentrations of PM2.5 measured in one home for just over 20 days. The raw sensor data has been adjusted by multiplying the raw sensor reading by 0.62, the value for gravimetric correction ratio based on unit collecting a filter of airborne fine particulate matter simultaneously as passing air through the light scattering sensor; i.e, comparing the gravimetric concentration based on the filter to the average of the raw sensor data over the entire deployment time period. [1]

The continuous PM_2.5_ sensor data was corrected by multiplying each value of a deployment by its gravimetric correction ratio. The distribution of gravimetrically corrected continuous sensor data for PM2.5 measured inside each home is shown in Fig. 1. The data displays the large range in 30 s average concentrations with many homes seeing repeated concentration peaks in the hundreds of µg/m^3^ and a few homes seeing average concentrations across the entire deployment period in the hundreds of µg/m^3^. An example time series plot in one home can be found in Fig. 2.

#### Household environmental triggers

As shown in Table 3, participants reported multiple sources of indoor PM_2.5_. Frequent use of gas stoves and frequent opening of windows facing major arterial roadways were common, each reported by more than 50% of participants. Incense or candle burning was reported by nine participants (30%), and exposure to environmental tobacco smoke (ETS) was reported by ten (33%). A significant association was observed between household PM_2.5_ levels and the use of aromatherapy products in the home (*p* = 0.007).

**Table 3:** Prevalence of environmental household triggers and its association with PM2.5 levels (*n* = 25)

Triggers	*N* out of 30 participants (%)	*N* out of 25 with ≥ 4 days of IAQ data (%)	Mean PM_2.5_ of unexposed group (µg/m^3^)	Mean PM_2.5_ of exposed group (µg/m^3^)	SD	IQR	U statistic^†^	*p*-value^†^
Frequent use of gas stove	19 (63)	15 (60)	116.77	48.43	25.46	39.7	74.00	0.956
Aromatherapy/scented candles/essential oil use	9 (30)	8 (32)	44.03	134.11	159.95	101.25	22.00	0.007*
Environmental Tobacco Smoke	10 (33)	8 (32)	83.01	56.15	39.76	42.25	66.00	0.907
Frequent window-opening facing major artery	16 (53)	15 (60)	98.21	59.57	54.53	62.2	71.00	0.824

*p < 0.05

^†^Mann-Whitney U Test

#### Post-monitoring interviews

Most participants (*n* = 21; 93%) found the IAQ sampling protocol acceptable. Concerns included potential surreptitious recording (*n* = 2; 7%) and minor device issues (*n* = 5; 17%). Receiving IAQ results was considered valuable by 24 participants (80%), and 20 (67%) reported study benefits such as increased awareness of asthma triggers, reduced smoking, and compensation. All but two participants (93%) would recommend the study to others with asthma. Six participants (20%) linked IAQ to climate change, while most (*n* = 16; 53%) associated it only to asthma control. Two individuals cited unconventional explanations for climate change.

### Post-trial protocol amendment

A post-trial protocol amendment enabled the study team to provide participants living in homes with high PM_2.5_ levels (i.e., > 35.5 µg/m³) with their results, low literacy educational materials, including a prototype infographic of their indoor PM_2.5_ levels, as well as free or low-cost strategies to improve indoor air quality, and references to local and state remediation resources. Of the 15 participants who had unhealthy household PM_2.5_ levels, 13 (87%) consented to receive their results. All 13 received the infographic and the educational/resource sheet and nine (60%) scheduled a counseling session about their results with the Principal Investigator. During these sessions, participants reported substantially higher exposure to indoor triggers than initially indicated on the environmental audit intake form.

Two participants with exceptionally high PM_2.5_ levels in their homes (> 200 µg/m³) were offered a second two-week UPAS v2 + sampling period, and both agreed. Both homes showed improvements in PM_2.5_ concentrations compared to the initial measurements. Although both homes continued to have unhealthy PM_2.5_ levels, one participant’s mean PM_2.5_ decreased from 212.0 µg/m³ to 69.0 µg/m³, while the second participant’s mean PM_2.5_ decreased from 505.6 µg/m³ to 161.5 µg/m³. One participant reported that the initial IAQ monitoring increased her sensitivity to environmental tobacco smoke (ETS) and led her to reduce secondhand smoke exposure in her home, a possible explanation for the reduced PM_2.5_ seen.

## Discussion

To the best of our knowledge, the indoor air quality of low-income households in NYC among Black adults with uncontrolled asthma has not been previously studied. This study demonstrated the feasibility of conducting IAQ monitoring in this population. Participants found the study procedures acceptable and wanted to know their IAQ results despite there being no plan to return results in the original feasibility trial. Our findings found high PM_2.5_ levels and exposure to environmental triggers in these homes, highlighting the need for larger studies focused on mitigating these hazards.

Our study participants had an average indoor PM_2.5_ level of 72.24 µg/m³, which is alarmingly high compared with the NYC average outdoor 24-hour PM_2.5_ level of under 35 µg/m³, as measured through a combination of street-level and rooftop monitors [43]. The higher-than-average PM_2.5_ levels observed in this study were anticipated, given prior research showing that high-poverty neighborhoods in NYC often experience increased health risks [44, 45], including PM_2.5_-attributable asthma emergency department visits 4.5 times higher in low-income neighborhoods compared to wealthier areas [46]. Pediatric asthma studies have previously demonstrated that elevated indoor PM_2.5_ exposure is associated with reduced peak expiratory flow rate, an increase in uncontrolled asthma and a higher number of asthma-related emergency visits [47–52], not unlike our participants who had uncontrolled asthma as measured by ACQ, a high degree of symptom burden as measured by self-reported rescue medication use and above average acute health care utilization.

In comparison, several studies have measured indoor PM_2.5_ in general adult populations with asthma, providing relevant context for interpreting exposure levels and their respiratory consequences. Balmes and colleagues enrolled 302 adults with asthma and/or rhinitis in the greater Baltimore area and found a mean indoor PM_2.5_ level of 10.8 ± 2.6 µg/m³ (sampled across three home locations), with notably higher concentrations in the kitchen (39.1 ± 107.3 µg/m³) and living room (40.5 ± 111.2 µg/m³); higher PM_2.5_ levels were associated with greater asthma symptom burden [53]. In an Italian study by Simoni and colleagues of 421 homes of individuals with asthma or asthma symptoms, PM_2.5_ was measured over one week each in summer and winter, with mean concentrations of 67 µg/m³ in 139 rural homes and 76 µg/m³ in 282 urban homes; asthma symptoms were associated with higher PM_2.5_ levels [54]. To the best of our knowledge, this is the first study to explore PM_2.5_ levels for durations greater than one week in the homes of urban, low-income Black adults with uncontrolled asthma.

Participants’ were exposed to numerous household environmental triggers, such as unvented gas stove use and environmental tobacco smoke, aligning with previous findings involving low-income housing and sociocultural practices contributing to indoor air pollution [55, 56]. A few studies in pediatric settings have highlighted certain household characteristics (e.g., fungi diversity, dust, stove type) that exacerbate asthma symptoms, suggesting a likely parallel in adult populations [48, 57–59]. By identifying significant associations between triggers and elevated particulate matter, our study extends the insights from children to adults.

National guidelines [60] and global expert reports [61] outline critical components of asthma self-management education that largely focus on individual behaviors such as the use of controller medication, monitoring and response to symptoms, and avoidance and remediation of allergic environmental factors. If further evidence confirms the impact of indoor air quality on uncontrolled asthma, these triggers could become new targets for intervention and education that clinical guidelines could address. Participants expressed strong interest in receiving their IAQ results, which can be leveraged to support better asthma self-management and informed decision-making through reducing in-home triggers. Educational programs aimed at increasing awareness of practical strategies to mitigate indoor air pollutants would be particularly valuable for this population. As a first step, we developed a handout with local and state IAQ resources designed for individuals with lower health literacy, along with a prototype infographic to be further developed using participatory design principles.

The success of conducting IAQ monitoring in this population underscores an opportunity for more comprehensive IAQ datasets, potentially providing more insight into IAQ’s role in disparities in adult asthma outcomes. Currently, there are consumer-grade low-cost indoor air quality monitors that allow for identifying air quality trends (i.e., increase in PM2.5 while cooking, or decrease in PM_2.5_ when using air purification), however IAQ monitors with filters that can provide real-time, gravimetrically corrected data are often costly [62, 63]. Future research could support policies that prioritize air quality standards in residential buildings such as requiring installation, maintenance, and monitoring of working kitchen ventilation systems. Findings from this study also suggest that there is a unique opportunity for public health stakeholders to collaborate with public housing stakeholders to address indoor air quality as an important social determinant of health. Cross-sector partnerships may lead to more focused public awareness campaigns on practical steps to improve IAQ for Black adults with asthma.

This study has several limitations. As a feasibility trial with only 30 participants, reported associations between household environmental triggers and PM_2.5_ should be interpreted cautiously. These triggers were self-reported, and participants were not able to specify the type of aromatherapy used, if any, which may have been useful in further identifying contributors to PM_2.5_ levels. Assessment of indoor triggers was also based on a single home inspection, which may not capture the temporal variability in exposures attributable to episodic indoor sources; repeated diary or ecological momentary assessment methods would better characterize the real-time occurrence of specific triggers. Selection bias is possible, as participants were recruited from the same pool as our parent trial and may have been more willing to engage in research and/or had higher levels of poor IAQ. Additionally, unexpected malfunctions of the UPAS v2 + units resulted in variability in the amount of data collected per participant. While these technological issues were quickly resolved, this may limit the reproducibility of the protocol in other settings. We also recognize that this research study included a majority of female participants, likely due to our recruitment from FQHCs and the higher prevalence of asthma among adult women. Our findings may not be generalizable to adult men with asthma.

## Conclusions

Our study demonstrated that collecting indoor air quality samples and characterizing household environmental triggers in NYC homes of low-income Black adults with uncontrolled asthma was both feasible and acceptable to participants. Future research should focus on larger cohort studies to establish more definitive associations between indoor air quality, household environmental triggers, asthma outcomes, and the impact of returning results along with actionable recommendations, as well as exploring the implementation of periodic IAQ monitoring by Medicaid and other insurers who would benefit from reducing emergency department visits due to poor IAQ.

## Supplementary Information

Supplementary Material 1.

Supplementary Material 2.

## Data Availability

The datasets used and/or analyzed during the current study are available from the corresponding author on reasonable request.

## Abbreviations

PM_2.5_: Fine particulate matter with diameters of ≤ 2.5 micrometers
IAQ: Indoor air quality
GHG: Greenhouse gas
NYC: New York City
ED: Emergency department
RCT: Randomized controlled trial
ACQ: Asthma Control Questionnaire
FQHC: Federally Qualified Health Center
UPAS v2+: Ultrasonic Personal Air Sampler, version 2+
ETS: Environmental Tobacco Smoke
SD: Standard deviation
NYCHA: New York City Housing Authority
EPA: Environmental Protection Agency
