# Supplementary material for: Assessing the feasibility of indoor air quality monitoring in low-income housing among Black adults with uncontrolled asthma in New York City
Source: BMC Public Health. 2026 Jul 8;26:2725. doi: 10.1186/s12889-026-28295-z (PMC13628984; doi:10.1186/s12889-026-28295-z)
Supplement: Supplementary file 1 [file 12889_2026_28295_MOESM1_ESM.docx]

**Supplemental Table 1. Objective and Self-Reported Assessment of Home Environment**

| **Item #** | **Item/Variable** | **Response format/options** |
| --- | --- | --- |
| **Home observations (completed by study staff)** | | |
| 1 | What kind of home does the research participant live in? | Single-select:  Apartment building (floor # _);  Brownstone;  Row house;  Single family home |
| 2 | Quantify the number of windows and label each window in the home/apartment as described below | For each street type: record # windows + room(s)  Main artery: # ___(which room(s) ___);  Minor artery: # ___(which room(s) ___);  Back street: # ___(which room(s) ___);  Wall or inner courtyard: # ___(which room(s) ___) |
| 3 | Can the kitchen be completely shut off from the rest of the living space (e.g., kitchen door, no pass-throughs in the walls)? | Yes / No |
| 4 | Does the kitchen have a ventilation system? | Yes (check all that apply):  Window(s);  Wall vent;  Air purifier;  Range hood;  No;  I don’t know; |
| 5 | Which of the following cooking appliances are in the home (select all that apply)? | Multi-select appliance type(s), each with status and fuel type  Oven: Working/Broken and Electric/Gas/Other/I don’t know  Stove/Range (combined stovetop and oven): Working/Broken and Electric/Gas/Other/I don’t know  Stovetop/Cooktop: Working/Broken and Electric/Gas/Other/I don’t know  None of the above  Other (specify___) |
| 6 | Measure the approximate distance between the participant’s cooking appliance and monitor | ___ feet |
| **Self-reported Questions (asked by the study staff to the research participant)** | | |
| 7 | How long have you lived in this home? | Open-ended: ___ |
| 8 | How many people live in the home? | Numeric entry: ___people |
| 9 | Do you have a humidifier? | Yes / No  If yes, typically, how often do you use your humidifier?  Several times a day;  Once a day;  Several times a week;  Once a week;  Never |
| 10 | If you do have a humidifier, answer the following Yes/No questions.  Is it automated?  Is it located in the living room?  Do you move it around? | Yes / No |
| 11 | Do you have an essential oil/aromatherapy diffuser in the home? | Yes / No |
| 12 | Do you have an essential oil/aromatherapy diffuser in the home? | Yes / No  If yes, typically, how often do you use your diffuser?  Several times a day;  Once a day;  Several times a week;  Once a week;  Never |
| 13 | If you do have a diffuser, answer the following Yes/No questions.  Is it automated?  Is it located in the living room?  Do you move it around? | Yes / No |
| 14 | If there is a kitchen door, does the kitchen door stay closed or open when household members cook? | Single-select:  Closed;  Open;  A mix;  Other (specify ___) |
| 15 | Typically, how often do you turn on your cooking appliance (for any reason)? Select options separately for oven, stovetop, cooktop, other cooking appliance (specify____) | Single-select:  Several times a day;  Once a day;  Several times a week;  Once a week;  Never |
| 16 | How many people in the home smoke cigarettes, e-cigarettes or other tobacco products? | Numeric entry: ___ people |
| 17 | If you selected at least one person for the last question, please fill out tobacco/nicotine product use details. | Grid-table entry by person who smokes (participant, spouse/partner, other) and product (cigarettes, cigars, pipes, e-cigarettes)  Specify the n that smokes each product; Amount per day;  Where you/they smoke (outside/in the home);  If inside, which room? |
| 18 | Are there children with asthma who live in the home? | Yes (how many ___) / No  If yes, do they still have active asthma (e.g., they see a health care professional for their asthma or take asthma medications)? Yes / No / Other (specify ___) |
| 19 | In the last week, how many hours a day you spend outside of the home? | Numeric entry: ___ hours/day |
| 20 | In the last week, where did you spend the remaining hours in a day? | Numeric entry for each location:  Living room ___;  Bedroom ___;  Kitchen/Dining ___;  Combined living and sleeping area ___ |
| 21 | In the last week, did you keep your windows open? | Single-select: Never;  Occasionally;  Often  If participants chose often or occasionally, how wide were the windows open?  Opened a crack (<4 inches);  Opened wide (>4 inches);  Other (specify___) |
