# Supplementary material for: Assessing the feasibility of indoor air quality monitoring in low-income housing among Black adults with uncontrolled asthma in New York City
Source: BMC Public Health. 2026 Jul 8;26:2725. doi: 10.1186/s12889-026-28295-z (PMC13628984; doi:10.1186/s12889-026-28295-z)
Supplement: Supplementary file 2 [file 12889_2026_28295_MOESM2_ESM.docx]

**Supplemental Table 2. Post-monitoring interview guide items**

**Experience in the study**

1. How well do you think the study went for you?

**Probes:** Did you come across any issues in the two weeks with the device? Did it get in the way of your ability to move around the house? Did household members or guests come over and ask questions? Do you think you could keep it in your home for a longer period of time than the two weeks? Did other family members have an issue with the device?

2. How much effort did you feel was required from you to participate in the trial?

**Probes:** Throughout the two weeks, did you devote any time to monitoring the device or checking on it? Did anyone else in the home monitor the device?

3. Did you understand what was expected from your participation in the study?

**Probes:** Was there anything you were worried about? Was there anything about the study and what you had to do that you were unclear about? Were there any instances throughout the two weeks where you had questions about the study? Did you contact anyone from the study team? If yes, were they able to answer your questions? Were you satisfied with the level of communication with the study team? Was it too frequent or not enough? Do you have any suggestions for improvement?

**Affective Attitude**

4. How did you feel about monitoring? Did you have any concerns?
5. Would you recommend someone with asthma to participate in this study?
6. Have you thought about climate change as it relates to your neighborhood, New York City, or the air quality in your home? Have you thought about how climate change or your indoor air quality may affect your asthma?
7. Can you tell me about the thing you liked most about being in this trial and the thing you liked least about being in the trial?
8. Based on your participation, did you feel like the compensation was an appropriate amount? Do you think you should have gotten money? Less money?

**Probes:** As we plan for a future trial, what do you think is an adequate amount of compensation for a patient to participate in this type of study? If we were to cut it for budget reasons, what is the lowest amount you would accept? What did you do/ plan with your incentive?

**Opportunity costs**

9. Did you find any benefits from participating in this study?
10. Were there any consequences (drawbacks) from participating in this study?

**Intention**

11. What were your reasons for participating?
12. How likely are you to participate in a similar intervention?
13. Do you have any other comments that you would like to share with us?
